# A new lineage of Galapagos giant tortoises identified from museum samples

**DOI:** 10.1038/s41437-022-00510-8

**Published:** 2022-02-25

**Authors:** Evelyn L. Jensen, Maud C. Quinzin, Joshua M. Miller, Michael A. Russello, Ryan C. Garrick, Danielle L. Edwards, Scott Glaberman, Ylenia Chiari, Nikos Poulakakis, Washington Tapia, James P. Gibbs, Adalgisa Caccone

**Affiliations:** 1 Department of Ecology and Evolutionary Biology, Yale University, New Haven, CT USA; 2 School of Natural and Environmental Sciences, Newcastle University, Newcastle Upon Tyne, UK; 3 MIT Media Lab, Massachusetts Institute of Technology, Cambridge, MA USA; 4 Department of Biological Sciences, MacEwan University, Edmonton, AB Canada; 5 Department of Biology, University of British Columbia, Kelowna, BC Canada; 6 Department of Biology, University of Mississippi, Oxford, MS 38677 USA; 7 Department of Life & Environmental Sciences, University of California, Merced, CA USA; 8 Department of Environmental Science and Policy, George Mason University, Fairfax, VA USA; 9 Department of Biology, George Mason University, Fairfax, VA USA; 10 Department of Biology, School of Sciences and Engineering, University of Crete, Irakleio, Greece; 11 The Natural History Museum of Crete, School of Sciences and Engineering, University of Crete, Irakleio, Greece; 12 Galapagos Conservancy, 11150 Fairfax Boulevard #408, Fairfax, VA 22030 USA; 13 University of Málaga, Campus Teatinos, Apdo. 59, 29080 Málaga, Spain; 14 Department of Environmental and Forest Biology, College of Environmental Science and Forestry, State University of New York, Syracuse, NY USA

**Keywords:** Phylogenetics, Population genetics

## Abstract

The Galapagos Archipelago is recognized as a natural laboratory for studying evolutionary processes. San Cristóbal was one of the first islands colonized by tortoises, which radiated from there across the archipelago to inhabit 10 islands. Here, we sequenced the mitochondrial control region from six historical giant tortoises from San Cristóbal (five long deceased individuals found in a cave and one found alive during an expedition in 1906) and discovered that the five from the cave are from a clade that is distinct among known Galapagos giant tortoises but closely related to the species from Española and Pinta Islands. The haplotype of the individual collected alive in 1906 is in the same clade as the haplotype in the contemporary population. To search for traces of a second lineage in the contemporary population on San Cristóbal, we closely examined the population by sequencing the mitochondrial control region for 129 individuals and genotyping 70 of these for both 21 microsatellite loci and >12,000 genome-wide single nucleotide polymorphisms [SNPs]. Only a single mitochondrial haplotype was found, with no evidence to suggest substructure based on the nuclear markers. Given the geographic and temporal proximity of the two deeply divergent mitochondrial lineages in the historical samples, they were likely sympatric, raising the possibility that the lineages coexisted. Without the museum samples, this important discovery of an additional lineage of Galapagos giant tortoise would not have been possible, underscoring the value of such collections and providing insights into the early evolution of this iconic radiation.

## Introduction

Remote oceanic islands and archipelagos have piqued the interest of evolutionary biologists for decades as these landscapes offer ideal settings to study colonization and subsequent establishment and diversification of species, largely owing to their isolation and discrete boundaries (Gillespie 2007). Moreover, island systems with well-known geological histories and ages of emergence (e.g., Hawaiian and Galapagos archipelagos) can also add a temporal axis to the study of the emergence of biological diversity (Cowie and Holland 2008; Parent et al. 2008; Shaw and Gillespie 2016).

The Galapagos Archipelago includes 13 major islands larger than 10 km^2^, six smaller islands, and over 200 islets and rocks, located in the Pacific Ocean ~900 km west of the South American continent and straddling the equator (Dirección del Parque Nacional Galápagos 2014). It is volcanic in origin, with islands formed by orogenetic activity along the Nazca plate, such that islands to the west are younger than islands to the east (Geist 1996; Geist et al. 2014). The complex history of the archipelago includes the islands drifting, merging and splitting, with some subsiding into the ocean (Ali and Aitchison 2014). This geological history impacted phylogeographic patterns of diverse native flora and fauna in some predictable ways (Parent et al. 2008; Grant and Grant 2014; MacLeod et al. 2015; Castañeda-Rico et al. 2019), including the Galapagos giant tortoises. Tortoises arrived on the oldest island and from there colonized the rest of the archipelago by a combination of active dispersal mediated by ocean currents, and vicariance closely matching the merging and splitting of islands (Poulakakis et al. 2012; Poulakakis et al. 2020).

**Fig. 1:**
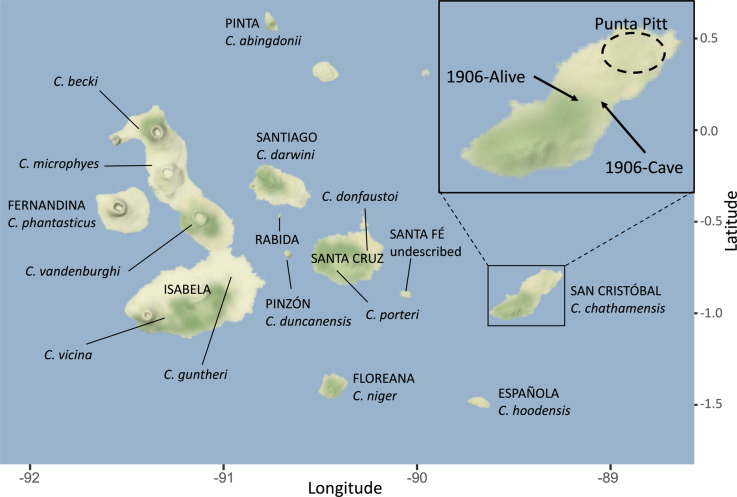
Map of the Galapagos Archipelago, indicating the locations of each *Chelonoidis* species, with San Cristóbal Island enlarged in the inset map. Island names are in capital letters. The approximate location of the cave where the bones were found in 1906 is marked, as is the approximate location where CAS 8133 was collected alive and the region of Punta Pitt.

Fourteen species of giant tortoise have been described from the Galapagos Archipelago, with one additional known but undescribed extinct species (Rhodin et al. 2017; Fig. 1). These species are genetically distinct with generally one species per island, except for the tortoises on Isabela and Santa Cruz Islands, where multiple species occur in allopatry (Caccone et al. 1999, 2002; Poulakakis et al. 2015). Three of the recognized species have gone extinct in the last century, and several others are in danger of extinction because of human impacts, including historical overharvesting and habitat degradation (IUCN 2020). These activities have reduced the number of tortoises on the archipelago to 10% of their historical population levels before humans arrived (Tapia et al. 2021).

In this study, we reconstruct the colonization and evolutionary history of Galapagos giant tortoises (*Chelonoidis* spp.) from San Cristóbal Island (formerly Chatham Island, Fig. 1), recognized as belonging to a single endemic species, *C. chathamensis*. Previous phylogenetic work has shown that the radiation of giant tortoises likely started from an initial colonization of San Cristóbal, the oldest emerged island of the archipelago, with founders sourced from western central South America (Caccone et al. 1999, 2002; Poulakakis et al. 2012, 2020). Accordingly, understanding past and present genetic differentiation of tortoises on San Cristóbal is crucial to understanding the history and pattern of diversification of Galapagos giant tortoises as a whole. San Cristóbal may have been part of a larger landmass 2–3 million years ago (MYA), however during times with higher sea levels its two volcanoes may have been separate islands, that have later re-coalesced into one island (Geist et al. 2014; Karnauskas et al. 2017). This dynamic geological history likely influenced connectivity among tortoises living on San Cristóbal over time. By extension, this might have impacted the colonization of the other islands.

Morphologically, adult Galapagos giant tortoises are characterized as possessing either saddleback or domed carapace shapes. This morphological diversity was recognized by early collectors (Van Denburgh 1914; Fritts 1983; Fritts 1984; Pritchard 1996) and more recently analyzed using geometric morphometrics (Chiari et al. 2009; Chiari 2021). The contemporary population of San Cristóbal tortoises, representing the entire species, *C. chathamensis*, has an intermediate carapace morphology that is highly variable among individuals (Chiari 2021).

On the island today there are ~6700 giant tortoises, having recovered from a bottleneck that reduced the population down to 500–700 individuals in the 1970s. Population recovery was achieved following the elimination of harvesting, removal of invasive species, and a short-lived but successful captive rearing program (Cayot 2008; Tapia et al. 2021). The contemporary population is found predominantly on the arid, northeastern part of the island. However, tortoises formerly resided on the more humid, southwestern and central parts of the island, where historical records suggest they were heavily harvested in the mid 1800s, leading to their extirpation by 1930 (Banning 1933). It was speculated that the southern tortoises represented a distinct species from the northern tortoises on the island (Pritchard 1996). Over time, a small number of tortoises have recolonized the southwestern and central parts of the island. Previous studies on a few samples from the contemporary population that were primarily collected in the northeastern part of the island, found no mitochondrial haplotypic diversity based on assaying a section of the typically highly variable non-coding mitochondrial control region (Caccone et al. 2002; Poulakakis et al. 2012). Previous studies have also not found evidence suggesting more than one genetically distinct taxon based on microsatellite data (Ciofi et al. 2002; Garrick et al. 2015).

In 1906, an expedition on San Cristóbal led by the California Academy of Sciences (CAS) collected a single living tortoise and found the disjointed remains of approximately 17 individuals that had evidently perished after falling into a cave located near the middle of the island (Fig. 1). Currently, pieces of six of these cave specimens are accessioned at the CAS (Van Denburgh 1914). While the single individual found alive had a domed carapace, the two intact carapace remains found in the cave were considered saddleback, although their morphological characteristics fall within the variation observed for intermediate shell shapes (see Chiari 2021). One of these cave specimens (CAS 8127) is the holotype of *C. chathamensis*, the endemic species from San Cristóbal.

Given the dynamic history of tortoises on San Cristóbal Island, and their pivotal role in underpinning the entire tortoise radiation, we analyzed historical samples and the contemporary population in detail. The objectives of this study were to (1) test the hypothesis that more than one lineage once lived on the island, and (2) describe patterns of genomic variation within the extant population. We collected mitochondrial control region sequence data from six historical samples and 129 samples from the contemporary population and compared these data to available sequences from all other extant and extinct Galapagos giant tortoise species to reconstruct their mitochondrial DNA evolutionary history. We further assessed the contemporary population for evidence of multiple lineages or admixture using two types of nuclear markers: a panel of 21 microsatellite loci and >12 000 single nucleotide polymorphisms (SNPs).

## Methods

### Sampling and data collection

#### Historical samples

Bone fragments were obtained from the California Academy of Sciences collections for six of the seven specimens collected from San Cristóbal Island in 1906: specimen CAS 8133 was collected alive, while the other five (CAS 8127 [long bone], CAS 8128 [long bone], CAS 8129 [long bone], CAS 8130 [skull], CAS 8131 [skull]) were collected from the cave (Fig. 1). One additional specimen collected from the cave in 1906 was not available for this study.

We used a Nikon XT H 225 ST to produce micro-computed tomography (micro-CT) scans of each bone specimen to locate the region with the highest density, and thus the best conditions for DNA preservation. Not all samples had a high-density region (e.g., CAS 8130 and 8131, the skulls), but where possible, we targeted the densest region. In a dedicated ancient DNA facility at Yale University, a Dremel rotary tool with a cutting blade was used to scrape off the surface of the bone, and then cut out a small wedge (~300 mg) for DNA extraction. The pieces of bone were powderized while submerged in liquid nitrogen using a Spex 6770 freezer mill. The bone powder was demineralized in a solution of 0.5 M EDTA pH 8.0, 10% SDS, and Proteinase K, that was incubated at 56 °C overnight in a shaking incubator. The resulting lysate was then mixed with 5x volumes of buffer PB (Qiagen) and centrifuged through a MinElute spin column (Qiagen) to bind the DNA. The MinElute column was washed twice using buffer PE. DNA was eluted from the column using 50 ul of ultra-pure water, warmed to 56 °C. All standard precautions to prevent contamination with contemporary DNA and between historical samples were followed.

We sequenced ~700 bp of the mitochondrial control region in four overlapping fragments using nested PCR and negative controls, as described in Poulakakis et al. (2008) on an ABI 3730 automated sequencer (Applied Biosystems). Multiple amplifications of each fragment were conducted and sequenced in both directions, which along with the overlapping regions of the fragments, gave multiple observations of each position along the sequence. Each position was closely inspected by eye to determine the consensus sequence for each individual in GENEIOUS (version 11.05; Kearse et al. 2012).

We also attempted to genotype the historical individuals at 12 microsatellite loci that have been successfully employed in previous studies of Galapagos giant tortoise museum samples using published protocols (Russello et al. 2007; Poulakakis et al. 2008; Russello et al. 2010), but in this case, they did not yield reliable genotypes.

A fragment of CAS 8128 was sent to the Center for Applied Isotope Studies at the University of Georgia for radiocarbon dating. The sample was mechanically cleaned using a scalpel and wire bristle brush to remove surface contamination and gently reduced to smaller fragments of approximately 3–5 mm in size. The bone fragments were demineralized in cold (4 °C) 1 N HCl for 24 h and rinsed with ultrapure water to neutral. The demineralized bone fragments were treated with 0.1 M NaOH at room temperature and rinsed with ultrapure water to neutral. The samples were rinsed with 1 N HCl to eliminate atmospheric CO_2_, rinsed in ultrapure water to pH 4 (slightly acidic), and heated at 80 °C for 8 h. The resulting solution was filtered through glass fiber filters to isolate the total acid insoluble fraction (“collagen”) and freeze-dried.

Approximately 1 mg of collagen was encapsulated in tin, and the elemental concentrations and stable isotope ratios (δ^13^C and δ^15^N) were measured using an elemental analyzer isotope ratio mass spectrometer (EA-IRMS). Values are expressed as δ13C with respect to PDB and δ15N with respect to AIR, with an error of less than 0.1%.

For accelerator mass spectrometry (AMS) analysis, a 5-mg subsample of collagen was combusted at 575 °C in evacuated and sealed Pyrex tubes in the presence of CuO to produce CO_2_. The CO_2_ samples were cryogenically purified from the other reaction products and catalytically converted to graphite using the method of (Vogel et al. 1984). Graphite ^14^C/^13^C ratios were measured using the CAIS 0.5 MeV AMS. Sample ratios were compared to the ratio measured from the Oxalic Acid I standard (NBS SRM 4990).

#### Contemporary samples

Blood (0.1–1.9 mL) was collected from live individuals during expeditions to San Cristóbal Island in 1999, 2012 and 2016. In 1999, samples were primarily collected from Punta Pitt on the northeastern point of the island (Fig. 1). In 2012, samples were collected from adults at the breeding center on San Cristóbal, some of which were originally found in Punta Pitt, while most were of unknown origin. The sampling in 2016 was conducted as part of a comprehensive census for tortoises on the entire island. In all cases, blood was stored in tubes containing a lysis buffer (Longmire et al. 1997) at ambient temperature in the field and at 4 °C upon arrival in the lab. Genomic DNA was extracted using a DNeasy Blood and Tissue Kit (Qiagen) following the manufacturer’s protocol for blood.

The same mitochondrial control region fragment targeted in the historical samples was newly sequenced (n = 72) or obtained from previous studies (*n* = 57) for 129 contemporary samples from San Cristóbal using previously described methods (Caccone et al. 1999).

For a subset of 60 contemporary individuals from the 129 sequenced for the control region, double digest Restriction-site Associated DNA (ddRAD) libraries were created following Peterson et al. (2012), as described in Miller et al. (2018), and sequenced using two lanes of Illumina HiSeq 2000 at the Yale Center for Genome Analysis. Sequences from these new libraries were combined with previously collected data for an additional 10 contemporary San Cristóbal individuals from Miller et al. (2018). These 70 individuals (10 sampled in 1999, 14 in 2006, and 46 in 2016) represent the geographic breadth of sampling locations across San Cristóbal Island. The sequences were processed and aligned to the *C. abingdonii* reference genome (assembly ASM359739v1, Quesada et al. 2019) using the PALEOMIX *bam pipeline* (version 1.2.14, Schubert et al. 2014). Briefly, we used PALEOMIX as a wrapper to efficiently implement read trimming using AdapterRemoval (version 2.3.1, Lindgreen 2012), alignment using BWA *mem* (version 0.7.17, Li and Durbin 2009), and indel realignment using GATK *IndelRealigner* (McKenna et al. 2010). Reads were then filtered to remove any with more than 4 mismatches to the reference using BAMTOOLS (version 2.5.1, Barnett et al. 2011). Variant detection and genotype calling were performed using BCFtools (Li et al. 2009) *mpileup* and *call*, excluding reads with a mapping quality score of less than 30, ignoring indels and outputting only variants. The resulting VCF file was filtered using VCFtools (Danecek et al. 2011) to exclude repetitive regions of the genome, sites with a sequencing read depth greater than one standard deviation above the mean depth (mean = 21.5, SD = 19.0), and sites with >20% missing data. Only biallelic SNP loci with a minor allele count of three were retained. Following these steps, we assessed missingness per individual, and removed six individuals with >50% missing data. Next, we re-filtered the original VCF file with only retained individuals, following the steps above, followed by a filter for Hardy-Weinberg Equilibrium (HWE) using the correction for false discovery rate described by Benjamini and Yekutieli (2001) (adjusted *p* value = 0.004888) and thinned the loci to retain one SNP per 10,000 bp in order to reduce linkage among SNP loci.

We genotyped these same 70 contemporary individuals from the ddRAD at 21 microsatellite loci, following the procedures described in Quinzin et al. (2019).

To summarize, in the analyses outlined below, a total of 6 historical and 129 contemporary mitochondrial control region sequences were used, and a subset of 70 of the 129 contemporary individuals were also genotyped at the nuclear microsatellites and SNPs (Supplementary Table 1).

### Genetic analyses

#### Phylogenetic analyses—mitochondrial DNA

To investigate phylogenetic relationships among the mitochondrial control region haplotypes found in the contemporary and historical individuals sampled from San Cristóbal, sequences were trimmed in GENEIOUS and aligned using Muscle (Edgar 2004) along with a representative subset of 28 unique haplotypes previously collected from all extinct and extant Galapagos giant tortoise species (Caccone et al. 2002; Beheregaray et al. 2003; Beheregaray et al. 2004; Caccone et al. 2004; Russello et al. 2005; Poulakakis et al. 2008; Chiari et al. 2009; Poulakakis et al. 2012; Edwards et al. 2013). The alignment was verified by eye, giving a final alignment length of 668 bp, and exported as a nexus file and opened in PopArt (http://popart.otago.ac.nz) where a statistical parsimony network (Templeton et al. 1992; Clement et al. 2000) was reconstructed.

To place the historical mitochondrial haplotypes within the Galapagos giant tortoise mtDNA-based phylogeny, we used a Bayesian approach implemented in BEAST2 (Bouckaert et al. 2019). The historical mitochondrial sequences from San Cristóbal tortoises were aligned with a more expansive dataset than for the network analysis, consisting of 93 haplotypes previously collected from all extinct and extant Galapagos giant tortoise species and the three outgroup species from continental South America (*Chelonoidis carbonarius*, *C. denticulatus* and *C. chilensis*) (Caccone et al. 2002, 2004), again using Muscle, this time with an alignment length of 718 bp due to gaps caused by alignment to the outgroup sequences. Genbank accession numbers are available in Supplementary Table 2. The BEAST input XML file was created using BEAUti v. 2.6.2. We used the BModeltest module of BEAST2 to select the best nucleotide substitution model and tested the four combinations of Birth-Death or Yule model for speciation and a relaxed log-normal or strict clock. Four chains of each analysis were run for 100 million Markov chain Monte Carlo (MCMC) replicates, logging the result every 1000 iterations, with a burn-in of 10%. We viewed the log files in TRACER v1.6 (Rambaut et al. 2018) to verify that convergence had been achieved and effective sample size (ESS) values >200 had been obtained for each chain. The tree and log files were combined across the four chains using LogCombiner v. 2.6.2, discarding a burn-in of 10% and maximum clade credibility trees annotated with the mean node heights were generated using TreeAnnotator v. 2.6.2. The combined log files were again viewed in TRACER, and the likelihood of the four models were compared using AICM (Baele et al. 2012).

#### Population genetic analyses—microsatellites and SNPs

We calculated genetic diversity metrics for the contemporary population’s nuclear marker datasets, including observed and expected heterozygosity and the inbreeding coefficient, G_IS_, using GENODIVE (Meirmans and Van Tienderen 2004). Effective population size (N_e_) was estimated using the bias-corrected method of linkage disequilibrium (Hill 1981; Waples 2006; Waples and Do 2010), implemented in NeESTIMATOR (Do et al. 2014), excluding alleles with a frequency below 0.05, with confidence intervals determined using the jackknife approach.

We used genotypic clustering analyses, STRUCTURE (Pritchard et al. 2000) and discriminant analysis of principal components (DAPC, Jombart et al. 2010), to evaluate evidence for substructure within the contemporary population. STRUCTURE was run using the admixture model with correlated allele frequencies, considering values of K (the number of clusters) ranging from one to 10, with 10 iterations per K. For the microsatellite dataset, each run included 1 million MCMC replicates following a burn-in of 100,000, whereas for the SNPs each run included 200 000 MCMC replicates after a burn-in of 100 000. To evaluate the support for each value of K, we plotted the log probability of the data (ln Pr(X | K)), and calculated the deltaK statistic (Evanno et al. 2005) using STRUCTURE HARVESTER (Earl and vonHoldt 2011). DAPC was implemented using adegenet (Jombart 2008) in R (R Development Core Team 2010). The best value of K was determined using the *find.clusters* function and based on the K with the lowest Bayesian information criterion (BIC) score. The optimal number of principal components to retain was found with the optim.a.score method, which was 1 for both datasets. We also used a principal components analysis to visualize clustering of genotypes, using the R package *Lea* (Frichot and François 2015).

## Results

### Phylogenetic analyses—mitochondrial DNA

**Fig. 2:**
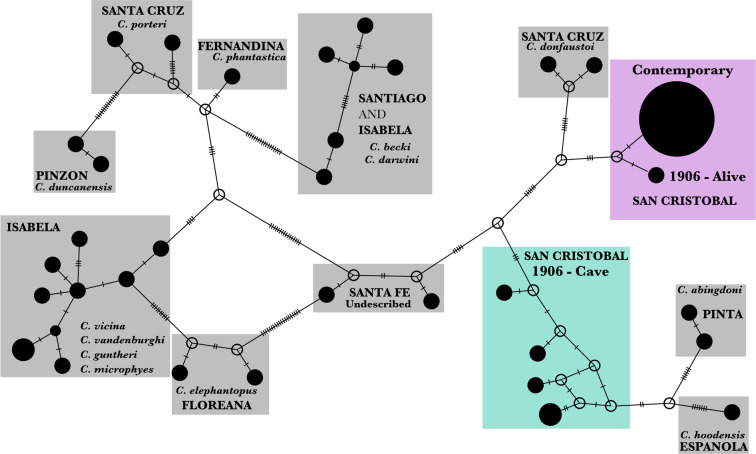
Statistical parsimony haplotype network of the mitochondrial control region (668 bp) for 129 contemporary individuals from San Cristóbal and six historical specimens collected in 1906, as well as 28 representative haplotypes from the other species of Galapagos giant tortoise. The name of the island where each species occurs is labeled with capital letters, with current taxonomy in italics. Haplotypes are represented as black circles on the network, the size of the circle is proportional to the frequency of the haplotype in the analysis. Open circles represent unsampled, hypothesized haplotypes, and hash marks indicate a single mutational change. Reticulations reflect uncertainty in relationships, or homoplasy.

A single mitochondrial control region haplotype was shared by the 106 contemporary individuals. None of the six individuals in the historical sample had this haplotype, but the domed individual sampled alive in 1906 (CAS 8133) had a haplotype that differed by only three substitutions from the contemporary haplotype. The five specimens found in the cave, including the type specimen (CAS 8127), had four novel haplotypes. These haplotypes formed a distinct cluster on the statistical parsimony network that links the haplotypes from *C. hoodensis* and *C. abingdonii* to the main network. All of the cave haplotypes are distinct from the contemporary and CAS 8133 haplotypes, being separated by 15–20 mutational steps (Fig. 2).

**Fig. 3:**
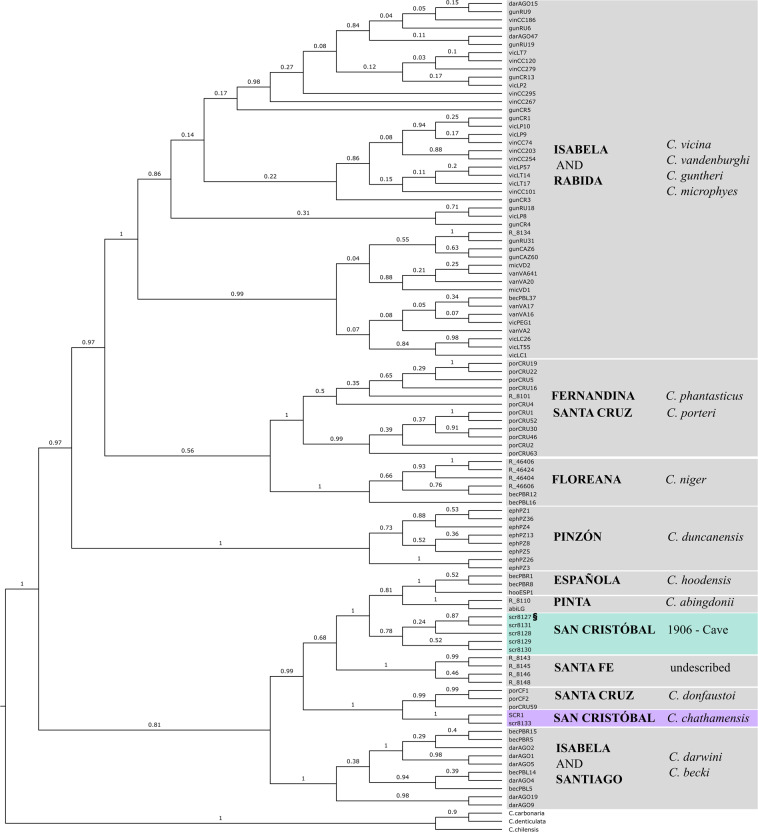
Bayesian Inference maximum clade credibility cladogram showing relationships among the San Cristóbal historical samples from the cave and collected alive in 1906, and a reference dataset of 93 Galapagos giant tortoise haplotypes and three outgroups based on the mitochondrial control region (alignment length 718 bp), estimated using BEAST with a strict clock and Birth Death tree. The numbers on the branches are the posterior probability support values. § indicates the *C. chathamensis* type specimen. The name of the island where each clade is found is in capitals, with current taxonomy in italics.

The Bayesian Inference phylogenetic analyses for the four parameter combinations each converged across the four chains, with combined ESS values greater than 900. The model comparison indicated that the parameter combination of the strict clock with a Birth Death tree had the lowest AICM value (Supplementary Table 3), and thus the best model fit, so this is the version we present and discuss, although the trees produced by the other models can be found in as Supplementary Fig. 1 A, B and C. The phylogeny (Fig. 3) included 93 representatives from all other species of Galapagos giant tortoise, the contemporary San Cristóbal haplotype, the six historical San Cristóbal individuals, and the three outgroup taxa. The San Cristóbal haplotypes were placed in the same main clade, along with the species *C. hoodensis*, *C. abingdonii*, *C. donfaustoi* and the undescribed species from Santa Fe. Within this main clade, the five specimens collected from the cave are in a clade sister to the one containing *C. abingdonii* and *C. hoodensis* (Fig. 3, posterior probability =1.0). As seen in the statistical parsimony network, the individual collected alive in 1906 (CAS 8133) belonged to the same clade as the contemporary San Cristóbal haplotype.

### Population genetic analyses—microsatellites and SNPs

Sequencing of the ddRAD libraries resulted in an average of 19.7 million reads per individual that aligned to the reference genome (range 5.6–87.9 million reads), which were combined with the 10 individuals sequenced previously (Miller et al. 2018). The final filtered ddRAD dataset had 64 individuals retained (six individuals were dropped due to high levels of missing data) that were genotyped at 12,192 loci with a mean depth of 16.5x, and 12% missing data. We omitted these six individuals from the microsatellite analyses in order to have fully overlapping datasets of the same 64 individuals. The microsatellite dataset of 21 loci had less than 1% missing data.

There were conflicting signals of heterozygote excess and deficit based on the type of nuclear marker assayed, with microsatellites showing a heterozygote deficit and SNPs showing heterozygote excess (Table 1). For both datasets, G_IS_ values were significantly different from zero based on 999 permutations, but in opposite directions (Table 1). However, for the microsatellite dataset, when calculating G_IS_ separately for each locus, none were significantly different from zero (Supplementary Table 4). The N_e_ value estimated from the microsatellites was higher than for the SNPs—34.2 versus 25.8, respectively, but the 95% jackknife confidence intervals were broad and overlapping (Table 1).

**Table 1:** Diversity measures within the contemporary population on San Cristóbal Island based on 21 microsatellite loci and 12 192 SNPs used to genotype the same 64 individuals.

MARKER	N_A_	EFFECTIVE N_A_	H_O_	H_E_	G_IS_	N_e_
SNPS	2	1.41	0.280	0.266	−0.053 (p = 0.001)	25.8 (18.2–38.0)
MICROSATELLITES	7.81	3.93	0.665	0.684	0.029 (p = 0.042)	34.2 (22.7–55.4)

G_IS_ p-values are based on 999 permutations. In parentheses for N_e_ are the 95% confidence intervals based on jackknifing.

N_A_, mean number of alleles per locus; Effective N_A_, the number of alleles that would be expected at a locus based on heterozygosity; H_O_, observed heterozygosity; H_E_, expected heterozygosity; G_IS_, inbreeding coefficient; N_e_, effective population size.

**Fig. 4:**
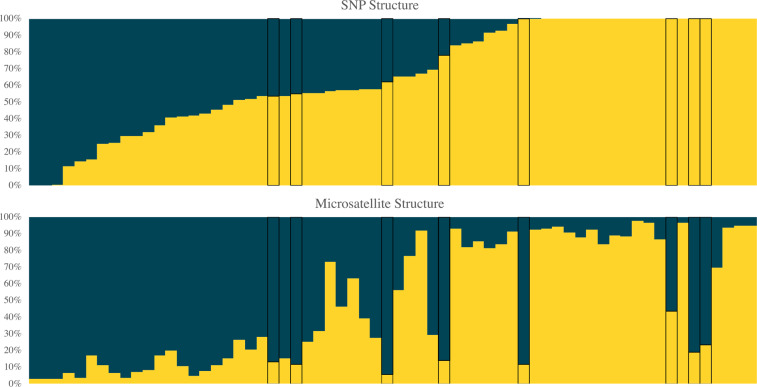
Barplots depicting K = 2 for the STRUCTURE analysis using the SNP (12 192 loci) and microsatellite (21 loci) genotypes for the contemporary San Cristóbal population (*n* = 64). Each bar represents an individual and the proportion of the bar that is each color represents the membership of that individual to the two clusters. The order of individuals is the same in both plots, black boxes around bars highlight individuals with a greater than 0.4 discrepancy in assignment proportions between the analyses.

The clustering analyses based on microsatellite and SNP genotypic data did not produce strong evidence for substructure within the contemporary San Cristóbal population. For STRUCTURE, K = 2 was suggested as most likely when examining the ln Pr(X | K) values (i.e. a large increase in ln Pr(X | K) between K = 1 and K = 2, but less substantial increases for larger values of K; Supplementary Fig. 2A and B) for both the microsatellite and SNP datasets. Although K = 2, 4 or 8 were considered likely by the deltaK statistic for the SNP data (Supplementary Fig. 2C), we were unable to estimate deltaK from the microsatellite analysis due to a lack of variance in ln Pr(X | K) values between iterations. In some cases, the microsatellite and SNP analyses strongly assigned the same individuals to alternate STRUCTURE clusters (Fig. 4), and in general there is a cline in membership between the two clusters. For DAPC, the BIC scores for K = 2 were just slightly lower than for K = 1, and density plots for the DAPC showed the two clusters as partially overlapping (Supplementary Fig. 3). The PCA did not reveal any distinct clustering of individuals (Supplementary Fig. 4). When the PCA is color coded according to STRUCTURE K = 2 cluster membership, there is a gradient along PC1, however this did not correspond to any identifiable geographic patterns when color coded by sampling region (Supplementary Fig. 4).

### Radiocarbon dating

CAS 8128 yielded 28.7% collagen, with an atomic carbon to nitrogen ratio of 3.4. The carbon 14 age was determined to be 410 years old, which corresponds to a calibrated date of 1450–1630 AD (95% probability) using the SHCal13 calibration curve (Hogg et al. 2013), as calculated in OxCal (Bronk Ramsey 2009).

## Discussion

### A new mitochondrial lineage of tortoises on San Cristóbal Island

Based on evidence from the mitochondrial control region, we have discovered a new lineage within the Galapagos giant tortoise radiation. All five of the skeletal remains collected in 1906 from the cave on San Cristóbal Island have haplotypes that are part of a clade that is distinct from the haplotype found in the contemporary population on the same island (Fig. 3). We evaluated mitochondrial control region sequences from 129 contemporary individuals collected across the island (~1.5% of the current estimated population on the island, Tapia et al. 2021), but found the same single *C. chathamensis* haplotype previously identified, suggesting that the “cave” lineage has gone extinct. Herein, we refer to this as the “extinct” San Cristóbal lineage. The sister clade to the extinct lineage is the one including the Pinta and Española species (*C. abingdonii* and *C. hoodensis*). Using the clade ages from Poulakakis et al. (2020), this would place the divergence between the extant and extinct lineages from San Cristóbal at around 0.72 MYA. These data (Fig. 3) clearly show that the extinct and contemporary lineages on San Cristóbal are not each other’s closest relatives based on their mitochondrial DNA, leaving their origin and relationship to the other Galapagos tortoise species unclear (Poulakakis et al. 2012, Poulakakis et al. 2020). At this point we can only formulate alternative hypotheses, some more likely than others, based on the known geology of the archipelago, the history of the group, and the data available. One possibility we can clearly dismiss is that the two mitochondrial DNA lineages derive from tortoises that colonized Galapagos from the continent at different times, as all the species in the Galapagos tortoise radiation are included in a single clade, separated from the continental species of *Chelonoidis*. A more likely scenario, following from our reconstructed phylogeny (Fig. 3), is that when tortoises initially dispersed from San Cristóbal to colonize the islands of Santiago, Santa Cruz, and Pinzón, there was only a single lineage on the island. This lineage may have subsequently split within San Cristóbal (perhaps due to high sea levels causing the island to be divided into two), with the ancestors of the extinct lineage colonizing Española, while the ancestors of the contemporary lineage colonized Santa Cruz, giving rise to *C. donfaustoi*. Alternatively, it could be that one of the two mitochondrial DNA lineages on San Cristóbal is derived from a colonization event from nearby islands, thus representing double colonization from two different sources, at different times (i.e., one continental, and subsequently, one intra-archipelago). However, we think that this is relatively unlikely, as the re-invasion would have occurred from islands to the West, such as Santa Cruz, while all studies so far support an East to West colonization pattern across the archipelago rather than West to East (Beheregaray et al. 2004; Poulakakis et al. 2012, 2020). A more likely scenario is that there was potentially a proto-island that included the two modern islands of Española and San Cristóbal when the first tortoises arrived from the continent (Poulakakis et al. 2012, 2020). On this island, tortoises might have differentiated into two different groups. Once the islands split, there could have been a recolonization event from Española to San Cristóbal, leading to the coexistence of two different taxa with different mitochondrial DNA lineages, with the one derived from the Española recolonization eventually going extinct, leaving as its only trace the mitochondrial DNA sequences retrieved from the cave bone remains. To evaluate the likelihood of these two possibilities we would need additional sequence data to provide more resolution as well as historical specimens from Española Island.

### Examining the contemporary population

It is unfortunate that we were unable to obtain microsatellite genotypes from the historical specimens, because assessing nuclear divergence will be critical to understanding the degree to which the extinct and contemporary taxa are distinct. Given the geographic proximity of the collection sites for the cave and live specimens in 1906 (Fig. 1), and the radiocarbon date of CAS 8128 being between 1450 and 1630 AD, it seems likely that the lineages were sympatric at least in recent history. Such a situation raises the possibility that the lineages come from two different gene pools that had fused, resulting in a single population with mitochondrial haplotypes from both ancestral lineages, that became fixed (or very nearly fixed) for the single haplotype following the subsequent bottleneck in the 1900s. Lineage fusion is documented to be occurring in the Galapagos giant tortoise species *C. becki*, where secondary contact between descendants of two separate colonizations of Volcano Wolf, both from Santiago Island, is resulting in introgressive hybridization (Garrick et al. 2014). The intermediate carapace morphology of the contemporary San Cristóbal population could be explained by this type of scenario, as elsewhere in the Galapagos, intermediate shell morphology is likely due to secondary contact among species with different carapace morphology (Fritts 1984; Russello et al. 2007; Poulakakis et al. 2008; Chiari 2021).

To evaluate evidence of possible admixture or substructure in the contemporary population that might be the legacy of past lineage fusion, we examined nuclear genetic markers in the contemporary population. Using both 21 microsatellites and >12 000 SNPs from ddRAD sequencing, we found some support for K = 2. This result, if reliable, would suggest the existence of two nuclear lineages on the island. However, evaluating the relative support for K = 1 and K = 2 can be difficult and it is important to interpret the results in light of the total evidence for genetic subdivision (Janes et al. 2017; Cullingham et al. 2020). In this case, the SNPs and microsatellites assigned some individuals with high membership to opposite clusters (Fig. 4), suggesting that there is not true signal to support the assignment. There is also no identifiable biologically meaningful pattern to the K = 2 clusters based on geography, illustrated by the PCA color coded by sampling region (Supplementary Fig. 4), where the single cloud of points has a random scattering of each color. Taken together, these results do not constitute strong evidence of population substructure that would support the continued existence of two nuclear lineages on the island.

The documented bottleneck down to an estimated 500–700 individuals on San Cristóbal in the 1970s has evidently led to the fixation of the contemporary population to a single mitochondrial control region haplotype based on our sequencing of over 125 individuals. Studies that have compared the levels of nuclear diversity among the Galapagos giant tortoise species have found San Cristóbal to have moderate diversity (Garrick et al. 2015; Miller et al. 2018). Compared to our findings here, Garrick et al. (2015) found higher expected heterozygosity (0.73 vs 0.68) and effective population size (68, 95% CI 32–4592 vs 34, 95% CI 23–55, Table 1). However, Garrick et al. (2015) results were based on 12 microsatellites genotyped for 19 individuals sampled in 1999 from Punta Pitt, whereas ours included 10 of those same individuals plus 54 tortoises sampled from a broader area. It is not possible to directly compare the SNP diversity measures calculated here with those from Miller et al. (2018) because that study identified variants across all species of Galapagos giant tortoise, thus including many loci that are not variable within the San Cristóbal population. The difference in G_IS_ calculated between the SNPs and microsatellites in this study is probably due to locus AGG68, which has only two alleles (Supplementary Table 4), the minor with a frequency of just 5%. When this locus is excluded, the G_IS_ from microsatellites does not significantly differ from zero (data not shown), and the G_IS_ value for the SNPs, although significant, is only slightly negative and close to zero.

The estimates of N_e_ for the current population on San Cristóbal are 26 and 34 for the SNPs and microsatellites, respectively. Although N_e_ estimates based on the linkage disequilibrium method have been shown to be fraught, especially when the assumptions of an ideal population are violated (Waples and Yokota 2007; Waples 2010), we think that the N_e_ estimates here are reliable because they are based on a sample size (*n* = 64) that is larger than the estimated N_e_ for both markers, a condition which provides some extra confidence in the estimate (England et al. 2006). This is also supported by findings from previous SNP analyses in the Pinzón species of giant tortoise (Jensen et al. 2018a). In that study it was found that N_e_ estimates based on sample sizes above 60 individuals were relatively consistent. The comparison of SNP-based N_e_ estimates in these two species offers some additional insights into the demographic history of the San Cristóbal tortoises. The N_e_ estimate for the contemporary Pinzón population (N_e_ = 59) (Jensen et al. 2018a) is larger than that for San Cristóbal (N_e_ = 24), despite the former species having experienced a much more severe 20th century bottleneck to just 250 individuals. If we use the N_e_ and bottleneck census size for the Pinzon and San Cristóbal species to calculate Ne:Nc ratios, we obtain dramatically different values (0.24 and 0.03, respectively), providing additional support for San Cristóbal having experienced an extremely pronounced bottleneck that led to the fixation of a single mitochondrial haplotype.

## Conclusions and future directions

Understanding the evolutionary history of giant tortoises on San Cristóbal Island may hold further clues to reconstructing the broader tortoise radiation across the Galapagos Archipelago. Due to the highly degraded nature of the bones found in the cave, we were unable to collect genotypic data from microsatellites in this study, despite having used micro-CT scans to identify the region of bone that should yield the best-preserved DNA. Future efforts using shotgun sequencing or capture approaches may be able to yield a full mitochondrial genome (e.g., Jensen et al. 2018b), reduced representation of nuclear SNP loci (e.g., Gauthier et al. 2020), or a low-coverage nuclear genome (e.g., Yao et al. 2020), which could provide critical information on the level of divergence between the extinct lineage and other recognized taxa, and shed light on the possibility of lineage fusion on San Cristóbal. Based on the data in hand, we can confidently conclude that there was a previously unrecognized mitochondrial lineage, but without nuclear genetic data, it is unclear whether this lineage persisted as a distinct entity until it’s extinction in the 20th century.

The recognition of an additional lineage of giant tortoise on San Cristóbal Island may have implications for the taxonomy of *C. chathamensis*. The holotype for *C. chathamensis* is one of the cave specimens (CAS 8127), which has a very distinct mitochondrial haplotype from the contemporary population, which goes by that name. If future evidence based on nuclear genetic markers confirms that the extinct lineage warrants species status, a new name and type specimen would be required to represent the lineage still living on San Cristóbal today.

This study also provides important lessons that go beyond their relevance for this particular group of organisms, highlighting the importance of including a diachronic perspective to reconstruct the evolutionary history of a group. We are aware that this is not always possible, as our inference of the evolutionary past is routinely inferred from contemporary samples only. This study shows what could have been missed if samples collected at two different time points were not included. Without the ability to analyze genetic data from bone samples found in the cave together with samples from the contemporary population on the same island, the new mitochondrial lineage would remain unknown. This in turn has opened doors to new research directions into the taxonomy, systematics, and evolutionary history of this iconic radiation.

## Supplementary information

Supplemental Materials

## Data Availability

The microsatellite Structure file and SNP vcf file are available on Dryad (doi:10.5061/dryad.xwdbrv1fp). The demultiplexed ddRAD fastq files are available on the NCBI SRA as BioProject PRJNA802698. The newly generated mitochondrial control region sequences are available from Genbank (Accession MT899437—MT899442).
