# Supplementary material for: A new lineage of Galapagos giant tortoises identified from museum samples
Source: Heredity (Edinb). 2022 Feb 25;128(4):261–70. doi: 10.1038/s41437-022-00510-8 (PMC8987048; doi:10.1038/s41437-022-00510-8)
Supplement: Supplementary file 1 — Supplemental Materials [file 41437_2022_510_MOESM1_ESM.docx]

**Supplemental Materials for: A new lineage of Galapagos giant tortoises identified from museum samples**

**Authors:** Evelyn L. Jensen, Maud C. Quinzin, Joshua M. Miller, Michael A. Russello, Ryan C. Garrick, Danielle L. Edwards, Scott Glaberman, Ylenia Chiari, Nikos Poulakakis, Washington Tapia, James P. Gibbs, Adalgisa Caccone

**
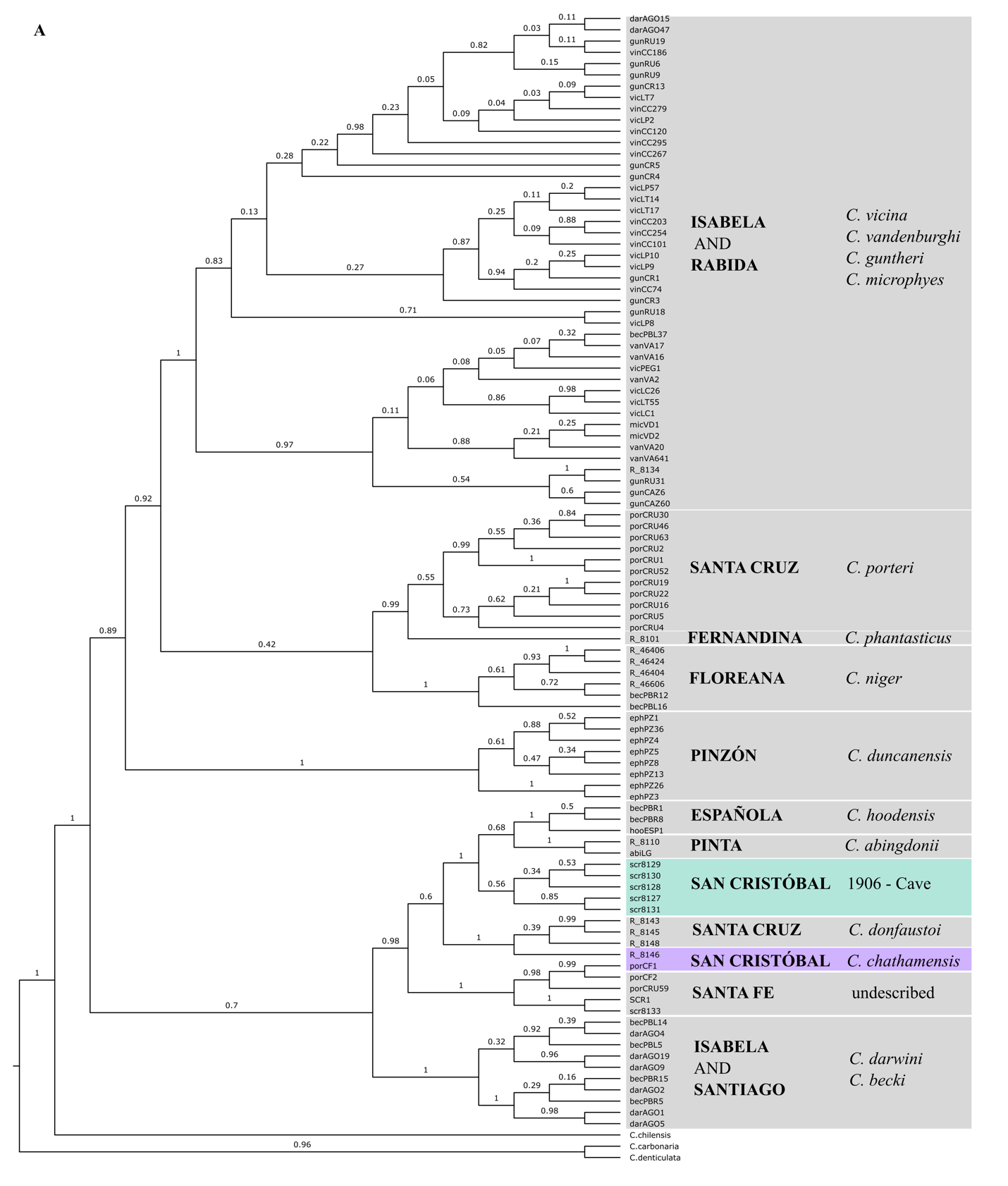
**

**
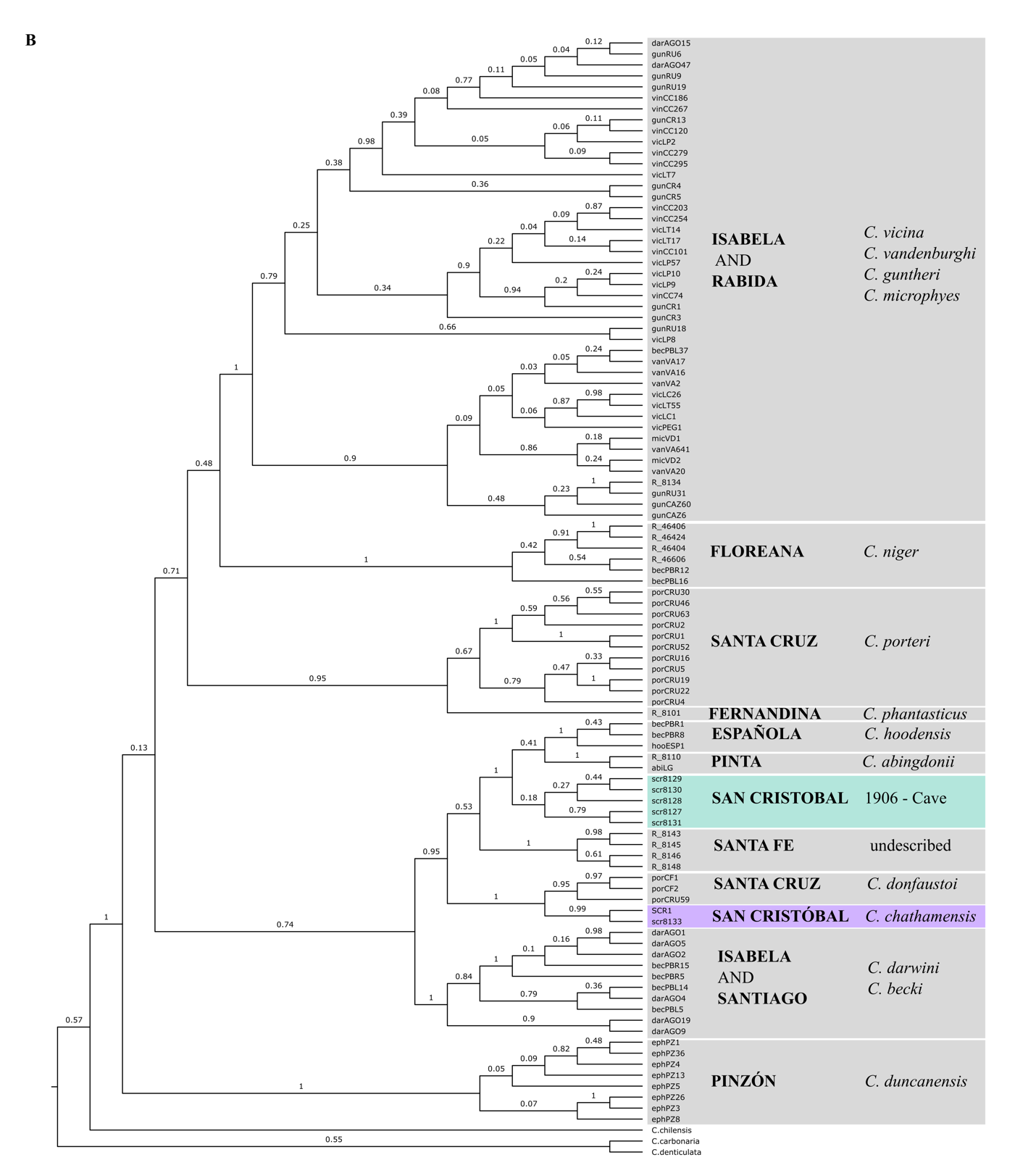
**

**
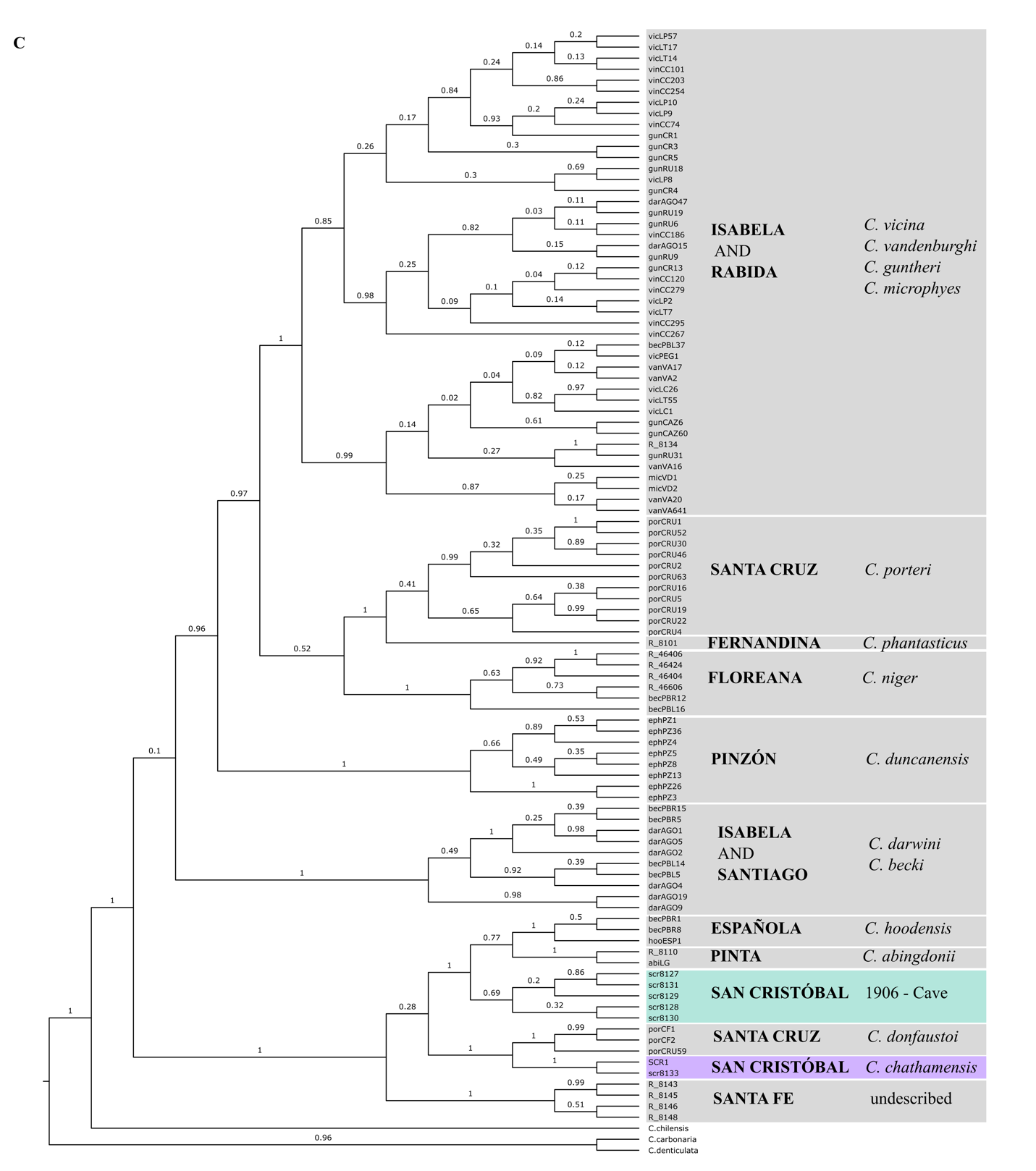
Supplemental Figure 1.** Bayesian Inference maximum clade credibility cladograms showing relationships among the San Cristobal historical samples from the cave and collected alive in 1906, and a reference dataset of 93 Galapagos giant tortoise haplotypes and three outgroups based on the mitochondrial control region (alignment length 718 bp), estimated using BEAST with A. a relaxed log-normal clock and Birth Death tree, B. a relaxed log-normal and Yule tree, and C. a strict clock and Yule tree. The numbers on the branches are the posterior probability support values. The name of the island where each clade is found is in capitals, with current taxonomy in italics.

Supplemental Figure 2. Plot of ln P(*K*) from the Structure analysis of A) the SNP and B) the microsatellites genotypic data and C) the values of DeltaK for the SNP based analysis.


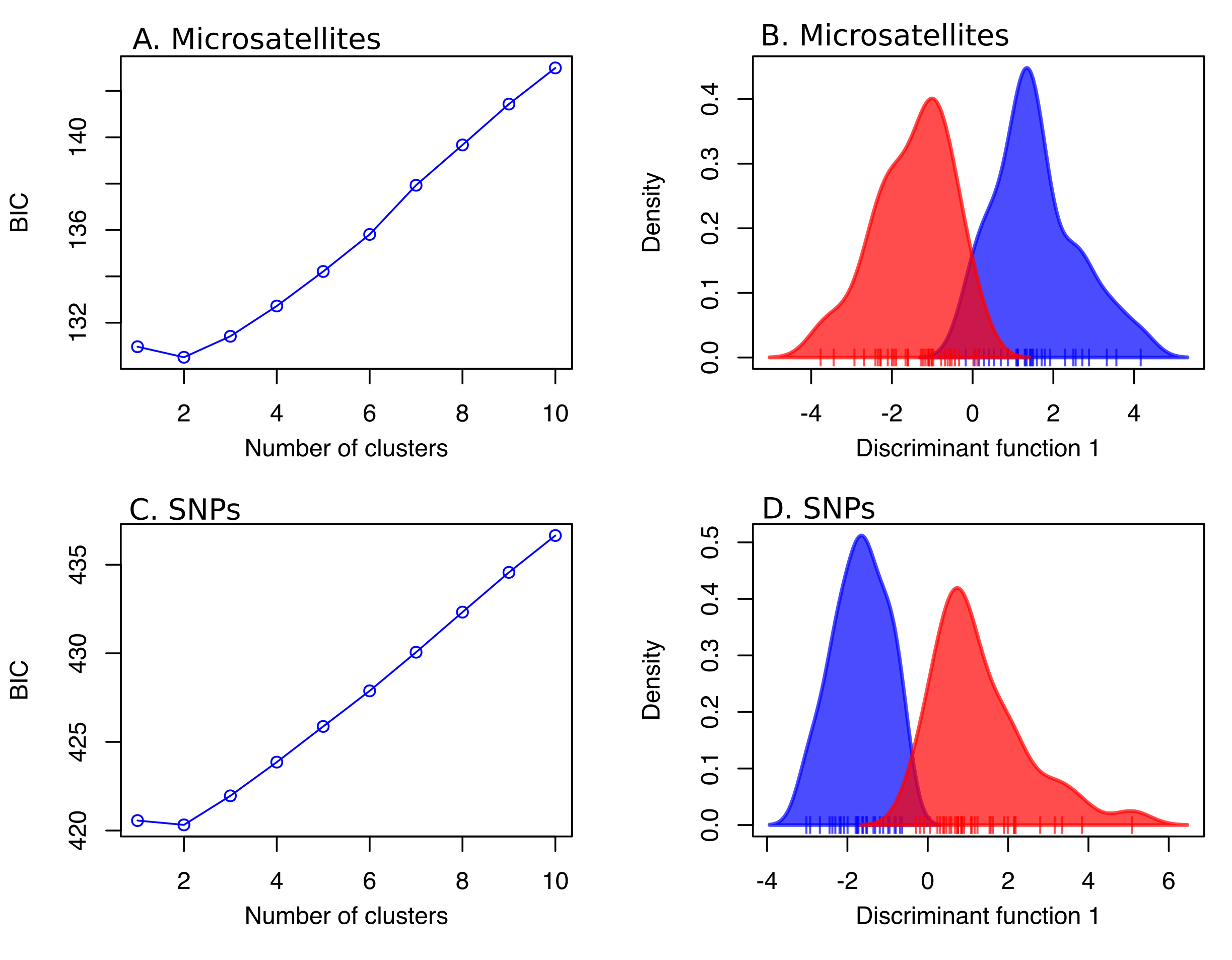


Supplemental Figure 3. Plot of Bayesian Information Criterion (BIC) from the Discriminant Analysis of Principal Components (DAPC) analysis of the contemporary San Cristobal population (n=64) depicting the support for each number of clusters evaluated for A) the microsatellite (21 loci) and C) the SNP (12 192 loci) genotypic data. Density plots showing the level of overlap between the clusters for B) the microsatellite and D) the SNP genotypic data.

Supplemental Figure 4. PCA of the 12 192 locus SNP dataset for the contemporary San Cristobal population (n=64) color coded by A) data collected in the present study versus by Miller et al (2018), illustrating that there is no batch effect due to data collected at different times being analyzed together; B) sampling location, where known; and C) structure cluster membership at K=2 based on the SNP dataset, with individuals with less than 70% membership to a single cluster considered to be mixed. For b, note that individuals sampled at the breeding center are of unknown origin, and “south” refers to any part of the island south of Punta Pitt. Each individual tortoise sampled is represented as a point, percentages correspond to the amount of variation explained by that axis.

Supplemental Table 1. Number of samples included for each marker type for each sampling period.

| Sample Period | Mitochondrial  Control Region | Microsatellites and SNPs |
| --- | --- | --- |
| Historical | 6 | - |
| 1999 | 20 | 10 |
| 2012 | 37 | 11 |
| 2016 | 72 | 43 |
| Total | **135** | **64** |

Supplemental Table 2. Genbank accession numbers for control region sequences. * indicates new sequences generated in this study.

| **Individual** | **Accession #** |
| --- | --- |
| abiLG | AY098029 |
| becPBL14 | AY098104 |
| becPBL16 | AF548280 |
| becPBL37 | AY098107 |
| becPBL5 | AF548255 |
| becPBR1 | AY098108 |
| becPBR12 | AF548283 |
| becPBR15 | AF548229 |
| becPBR5 | AY098110 |
| becPBR8 | AF548282 |
| darAGO1 | AY097959 |
| darAGO15 | AF548284 |
| darAGO19 | AF548254 |
| darAGO2 | AY097961 |
| darAGO4 | AY097963 |
| darAGO47 | AF548285 |
| darAGO5 | AY097964 |
| darAGO9 | AY097968 |
| ephPZ1 | AY098053 |
| ephPZ13 | AF548213 |
| ephPZ26 | AF548214 |
| ephPZ3 | AY098057 |
| ephPZ36 | AF548215 |
| ephPZ4 | AY098058 |
| ephPZ5 | AY098060 |
| ephPZ8 | AY098064 |
| gunCAZ6 | AF548257 |
| gunCAZ60 | AY097972 |
| gunCR1 | AF548236 |
| gunCR13 | AF548235 |
| gunCR3 | AF548232 |
| gunCR4 | AF548234 |
| gunCR5 | AY097993 |
| gunRU18 | AF548261 |
| gunRU19 | AF548263 |
| gunRU31 | AF548262 |
| gunRU6 | AF548259 |
| gunRU9 | AF548260 |
| hooESP1 | AY098010 |
| micVD1 | AF548230 |
| micVD2 | AY098098 |
| porCF1 | AY097977 |
| porCF2 | AY956612 |
| porCRU1 | AY097997 |
| porCRU16 | AY098070 |
| porCRU19 | AF548221 |
| porCRU2 | AY097998 |
| porCRU22 | AF548222 |
| porCRU30 | AF548223 |
| porCRU4 | AY098000 |
| porCRU46 | AF548224 |
| porCRU5 | AY098001 |
| porCRU52 | AF548225 |
| porCRU59 | AF548227 |
| porCRU63 | AF548226 |
| R-46404 | EU888933 |
| R-46406 | EU888934 |
| R-46424 | EU888943 |
| R-46606 | AY956613 |
| R-8101 | JN637234 |
| R-8110 | JN637232 |
| R-8134 | JN637233 |
| R-8143 | JN637236 |
| R-8145 | JN637237 |
| R-8146 | JN637235 |
| R-8148 | JN637238 |
| SCR1 | AY098072 |
| scr8127 * | MT899437 |
| scr8128 * | MT899438 |
| scr8129 * | MT899439 |
| scr8130 * | MT899440 |
| scr8131 * | MT899441 |
| scr8133 * | MT899442 |
| vanVA16 | AF548267 |
| vanVA17 | AF548264 |
| vanVA2 | AF548268 |
| vanVA20 | AF548265 |
| vanVA641 | AY098085 |
| vicLC1 | AY098025 |
| vicLC26 | AF548243 |
| vicLP10 | AY098030 |
| vicLP2 | AF548237 |
| vicLP57 | AF548241 |
| vicLP8 | AF548240 |
| vicLP9 | AF548239 |
| vicLT14 | AF548244 |
| vicLT17 | AF548246 |
| vicLT55 | AY098035 |
| vicLT7 | AF548245 |
| vicPEG1 | AF548248 |
| vinCC101 | AF548270 |
| vinCC120 | AF548271 |
| vinCC186 | AF548272 |
| vinCC203 | AF548273 |
| vinCC254 | AF548274 |
| vinCC267 | AF548275 |
| vinCC279 | AF548276 |
| vinCC295 | AF548277 |
| vinCC74 | AY097975 |
| *C. carbonaria* | AF351780 |
| *C. chilensis* | AF346029 |
| *C. denticulata* | AF346028 |

Supplemental Table 3. AICM values for the four parameter combinations of Birth Death or Yule tree, and strict or relaxed log-normal clock in the Bayesian Inference phylogenetic analysis, as calculated using the model comparison function in TRACER v1.6.

| Parameter combination | AICM | Standard error |
| --- | --- | --- |
| Birth death, Relaxed log-normal | 7062 | 0.18 |
| Birth death, Strict | 7014 | 0.15 |
| Yule, Relaxed log-normal | 7056 | 0.13 |
| Yule, Strict | 7138 | 0.28 |

Supplemental Table 4. Diversity measures for each of the 21 microsatellite loci calculated within the contemporary population on San Cristóbal.

| Locus | N_A_ | Effective N_A_ | H_O_ | H_E_ | G_IS_ |
| --- | --- | --- | --- | --- | --- |
| AC039 | 5 | 2.692 | 0.641 | 0.633 | -0.011 |
| AC190 | 10 | 4.549 | 0.906 | 0.785 | -0.154 |
| AC149 | 5 | 2.952 | 0.571 | 0.667 | 0.144 |
| AC247 | 17 | 5.211 | 0.766 | 0.815 | 0.06 |
| AC251 | 7 | 4.531 | 0.889 | 0.785 | -0.133 |
| AGG68 | 2 | 1.1 | 0 | 0.092 | 1 |
| GAL21 | 7 | 3.618 | 0.688 | 0.73 | 0.058 |
| GAL158 | 4 | 1.599 | 0.286 | 0.378 | 0.245 |
| GGA45 | 3 | 1.868 | 0.444 | 0.469 | 0.052 |
| GAL45 | 12 | 5.45 | 0.828 | 0.823 | -0.006 |
| Gal75 | 7 | 4.078 | 0.75 | 0.761 | 0.014 |
| Gal263 | 15 | 6.365 | 0.672 | 0.851 | 0.21 |
| Gal50 | 16 | 8.095 | 0.859 | 0.884 | 0.027 |
| Gal159 | 5 | 1.828 | 0.469 | 0.456 | -0.027 |
| Gal100 | 7 | 2.975 | 0.688 | 0.669 | -0.028 |
| Gal94 | 4 | 2.847 | 0.688 | 0.654 | -0.052 |
| Gal127 | 10 | 5.047 | 0.984 | 0.807 | -0.22 |
| Gal136 | 7 | 4.582 | 0.578 | 0.79 | 0.268 |
| Gal194 | 7 | 3.685 | 0.766 | 0.734 | -0.043 |
| Gal288 | 8 | 5.337 | 0.688 | 0.82 | 0.162 |
| AC063 | 6 | 4.139 | 0.797 | 0.764 | -0.043 |

N_A_, mean number of alleles per locus; Effective N_A_, the number of alleles that would be expected at a locus based on heterozygosity; H_O_, observed heterozygosity; H_E_, expected heterozygosity; G_IS_, inbreeding coefficient
